# The Spatial Dynamics of Dengue Virus in Kamphaeng Phet, Thailand

**DOI:** 10.1371/journal.pntd.0003138

**Published:** 2014-09-11

**Authors:** Piraya Bhoomiboonchoo, Robert V. Gibbons, Angkana Huang, In-Kyu Yoon, Darunee Buddhari, Ananda Nisalak, Natkamol Chansatiporn, Mathuros Thipayamongkolgul, Siripen Kalanarooj, Timothy Endy, Alan L. Rothman, Anon Srikiatkhachorn, Sharone Green, Mammen P. Mammen, Derek A. Cummings, Henrik Salje

**Affiliations:** 1 Armed Forces Research Institute of Medical Sciences, Bangkok, Thailand; 2 Faculty of Public Health, Mahidol University, Bangkok, Thailand; 3 Queen Sirikit National Institute of Child Health, Bangkok, Thailand; 4 Department of Infectious Diseases, State University of New York, Syracuse, New York, United States of America; 5 University of Rhode Island, Providence, Rhode Island, United States of America; 6 Department of Medicine, University of Massachusetts Medical School, Worcester, Massachusetts, United States of America; 7 Center for Infectious Disease and Vaccine Research, University of Massachusetts Medical School, Worcester, Massachusetts, United States of America; 8 Department of Epidemiology, Johns Hopkins Bloomberg School of Public Health, Baltimore, Maryland, United States of America; University of North Carolina at Chapel Hill, United States of America

## Abstract

**Background:**

Dengue is endemic to the rural province of Kamphaeng Phet, Northern Thailand. A decade of prospective cohort studies has provided important insights into the dengue viruses and their generated disease. However, as elsewhere, spatial dynamics of the pathogen remain poorly understood. In particular, the spatial scale of transmission and the scale of clustering are poorly characterized. This information is critical for effective deployment of spatially targeted interventions and for understanding the mechanisms that drive the dispersal of the virus.

**Methodology/Principal Findings:**

We geocoded the home locations of 4,768 confirmed dengue cases admitted to the main hospital in Kamphaeng Phet province between 1994 and 2008. We used the phi clustering statistic to characterize short-term spatial dependence between cases. Further, to see if clustering of cases led to similar temporal patterns of disease across villages, we calculated the correlation in the long-term epidemic curves between communities. We found that cases were 2.9 times (95% confidence interval 2.7–3.2) more likely to live in the same village and be infected within the same month than expected given the underlying spatial and temporal distribution of cases. This fell to 1.4 times (1.2–1.7) for individuals living in villages 1 km apart. Significant clustering was observed up to 5 km. We found a steadily decreasing trend in the correlation in epidemics curves by distance: communities separated by up to 5 km had a mean correlation of 0.28 falling to 0.16 for communities separated between 20 km and 25 km. A potential explanation for these patterns is a role for human movement in spreading the pathogen between communities. Gravity style models, which attempt to capture population movement, outperformed competing models in describing the observed correlations.

**Conclusions:**

There exists significant short-term clustering of cases within individual villages. Effective spatially and temporally targeted interventions deployed within villages may target ongoing transmission and reduce infection risk.

## Author Summary

Transmission of dengue virus has long been studied in Kamphaeng Phet, Northern Thailand, but how cases are related in time and space is still unclear, as is the role of human movement in generating these patterns. Because of these knowledge gaps, public health officials cannot make educated decisions on how to target vector control interventions and mechanisms of virus dispersal are not known. We mapped the homes of dengue cases admitted to the main hospital in the province capital from 1994–2008 and quantified the spatial correlation between them. We found an almost three times greater chance that cases from the same month came from the same village than expected, given the overall distribution of cases. Some clustering was also observed between cases in neighboring villages with the overall epidemics experienced by neighboring communities also more correlated than epidemics in villages farther apart. The short-term clustering observed within individual villages implies that effective spatially targeted interventions deployed within villages may reduce infection risk. As the distance between neighboring communities exceeds the typical flight range of the dengue vector, these findings also suggest a potential role for human movement in driving the wider spread of the virus.

## Introduction

Dengue remains a major public health concern throughout global tropical and subtropical regions. An estimated 390 million people are infected by the mosquito-borne virus each year, of which 96 million develop symptomatic disease [1]. Thailand, like most countries in Southeast Asia, has experienced endemic dengue circulation of all four serotypes for decades [2], [3]. An effective dengue vaccine remains elusive and intervention measures will continue to rely on mosquito control for the foreseeable future. These efforts include the detection and removal of potential oviposition sites, the spraying of insecticides, and potentially the future releases of Wolbachia-infected mosquitoes that have been shown to reduce the mosquitoes' ability to transmit dengue [4]. Effective use of these measures requires a good understanding of the spatial distribution of cases. Of particular use is an understanding of where other cases are likely to be found on detection of an index case. Characterizing the spatial dependence between dengue cases can also provide insight into potential mechanisms of disease spread.

The home locations of individuals hospitalized with dengue in Bangkok have been shown to exhibit significant spatial dependence at distances of around a kilometer [5]. Such spatial structure suggests focal transmission events are driving viral dispersal in this large, super-urban population. The situation in rural areas, which make up the majority of the country, may be markedly different. Phylogenetic studies have shown widespread genetic and serotype diversity across the rural Thai province of Kamphaeng Phet with some clustering of lineages within villages [6], [7]. In addition, cluster studies in the same region detected infected individuals within 15 days of an index case at distances of 100 m within villages [8], [9]. However, the extent at which spatial dependence is observed in these areas is not known. Unlike continuously inhabited urban centers such as Bangkok, rural communities in Thailand tend to be separated by wide expanses of uninhabited farmland or forests. The distance between neighboring rural communities is typically far beyond the short flight range of the main dengue vector, *Aedes aegypti* [10]. For sustained transmission to occur between rural communities, movement of infected individuals is likely necessary. If human movement between neighboring communities were key to DENV dispersal in this region, we would expect short-term spatial dependence between cases occurring at between-community scales. Further, we would expect that patterns of population flows would correlate with the spatio-temporal location of infections. It has previously been shown that individuals tend to move to larger and closer communities [11]–[13]. Such population flows can be captured using gravity models that incorporate the size of populations and the distance between them. Similar approaches have previously been used in phylogenetic analyses to describe dengue viral flow in Vietnam [14]–[16].

Appropriate data necessary to describe the spatio-temporal patterns of dengue virus require, 1) a long time series, 2) availability of address data for patients, and proper diagnostics to confirm DENV infection. We used a unique dataset that meets all of these criteria: the geocoded home addresses of 4,768 individuals who were admitted to the provincial hospital in Kamphaeng Phet, Thailand over a fourteen-year period (1994–2008). The objective of our study was to characterize the short-term spatial dependence between dengue cases, to quantify the correlation in the long-term epidemics experienced by different communities and to explore the ability of human movement models to describe the observed correlations.

## Materials and Methods

### Ethics statement

Data were collected from existing records without personal data. The research components of this project received approval from the Ethical Research Committee of Faculty of Public Health, Mahidol University and U.S. Army Medical Research Materiel Command (USAMC-AFRIMS Scientific Review Committee) review and approval.

### Study area and data collection

**Figure 1:**
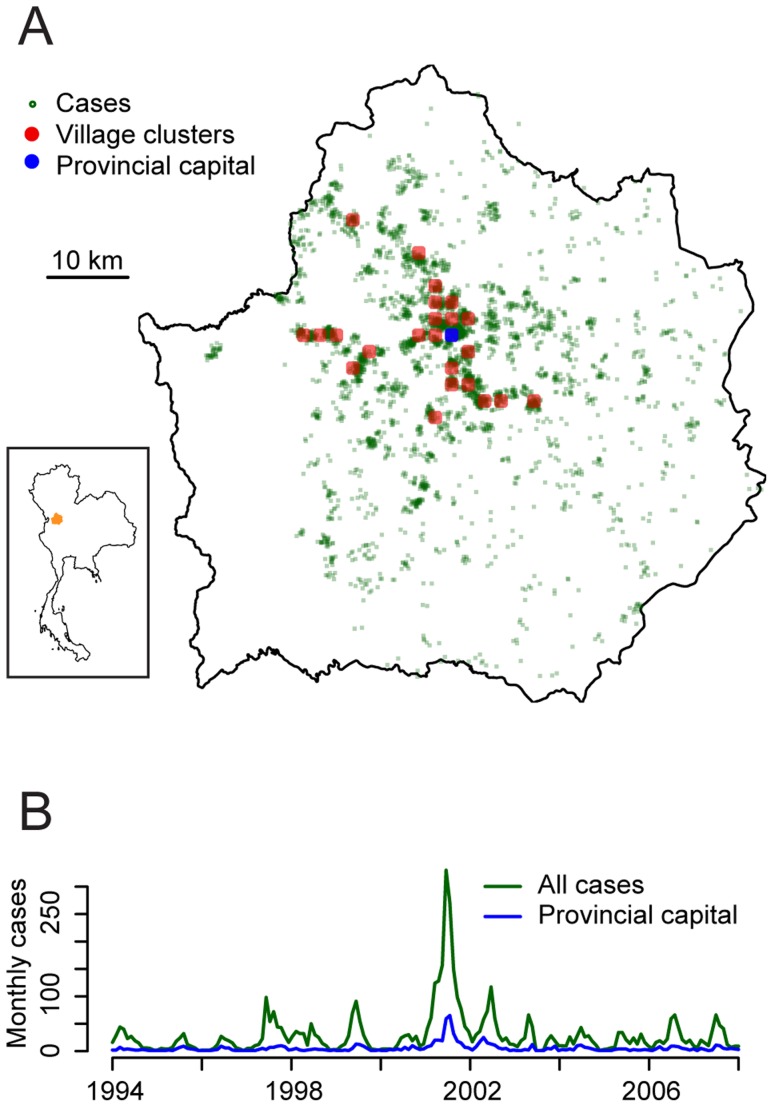
Spatial and temporal distribution of cases that presented at KPPH (1994–2008). (A) Map of case locations. The red circles mark the village clusters with at least 40 cases. (B) Total number of cases per month.

Kamphaeng Phet is a largely rural province in northern Thailand with an area of 8,600 km^2^ (Figure 1) [17]. It had a population of 797,000 people in the 2010 census, mainly residing in villages. The largest town in the province is the capital (Mueang Kamphaeng Phet) with 30,000 inhabitants. The landscape is dominated by rolling hills with large portions of the province covered by forests. Since 1994, the Armed Forces Research Institute of Medical Sciences (AFRIMS) has conducted dengue surveillance at Kamphaeng Phet Provincial Hospital (KPPPH). KPPH is the largest hospital in Kamphaeng Phet, located in the capital, and such receives referral cases as well as walk-in patients of all ages from throughout the province. For each suspected dengue case, DENV infection is confirmed using semi-nested RT-PCR and IgM/IgG ELISA. In addition, home address information is collected on each patient. We geocoded the home address down to the village level for each individual using detailed base maps of the region. Individuals from the same village were given the same coordinates (Table 1).

**Table 1:** Population characteristics.

Number of patients	5140
Mean age (years)	11.0
Hemorrhagic fever	3015 (59%)
Successfully geocoded	4768 (93%)

Population characteristics of patients admitted to Kamphaeng Phet Provincial Hospital between 1994 and 2008.

### Short-term spatial dependence between cases

To characterize the short-term spatial dependence between rural dengue cases, we used the 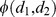 statistic on all cases occurring outside the provincial capital [5]. This statistic estimates the probability of two cases occurring both within distances *d_1_* and *d_2_* and within a month of each other relative to the independent probabilities of observing two cases within *d_1_* and *d_2_* over the entire time series and of observing two cases within a month of each other over the whole study area. This approach therefore measures the interaction in time and space of cases and has previously been used to characterize the spatial dependence of dengue cases in Bangkok [5].
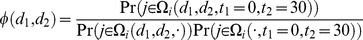
Where 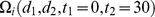 is the set of cases that occur both within a 30 day period and within *d_1_* and *d_2_* of case *i*; 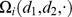 is the set of cases within *d_1_* and *d_2_* of case *i* over the entire time series and 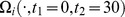 is the set of cases that occur within a 30 day period from case *i* over the study area. Importantly, as underlying spatial biases such as population density and hospital utilization rate differences impact both the numerator and the denominator in the same way, they do not bias our estimates of spatial dependence. We estimate 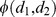 as follows (see [5] for details):
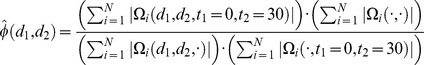
We generated bootstrapped confidence intervals for 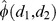 by resampling the cases with replacement 500 times. Ninety-five percent confidence intervals were calculated from the 2.5% and 97.5% quantiles from the resulting distribution. Patterns of spatial dependence may have changed over the time period of the study. We therefore recalculated 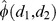 using cases from annual incremental five-year windows from between 1994 and 2008.

### Correlation between village clusters

We explored whether any short-term spatial dependence between individual cases resulted in correlation in the epidemics experienced by different communities. In this analysis, to avoid excessively small numbers of cases per location over the entire time period, villages were grouped into clusters by placing a grid over the province. The distance between each grid point was 3 km and villages were assigned to the closest grid point. Only village clusters with at least 40 cases over the time series were used in the analysis. The population of each village cluster was extracted from LandScan data [18]. LandScan uses a combination of satellite imaging and census data to construct population estimates throughout the world.

To make the epidemic curves between locations as comparable as possible, we down-sampled each epidemic curve (to create “down-sampled curves”) by randomly selecting 40 cases (the minimum number of cases at within a village cluster) with replacement from all the cases that occurred at that location. We calculated the Pearson correlation coefficient between all pairs of down-sampled curves. We calculated the loess curve of the relationship between the Euclidean distance and correlation between village cluster pairs. We repeated the down-sampling process 500 times and reported the mean of the resulting distribution. In addition, 95% confidence intervals for the loess curves were estimated from the 2.5% and 97.5% quantiles.

We compared our estimate of the expected correlation by distance separating communities to a theoretical complete-synchrony scenario where there was no distance effect. The complete-synchrony distribution was generated by randomly reassigning the location of all cases, keeping the month in which they occurred fixed. The total number of cases within any location over the whole time series was unchanged. The resulting distribution is that expected under a scenario of complete synchrony of cases over the province. The mean and confidence intervals for the complete-synchrony distribution were calculated by repeating the process above in generating down-sampled curves, repeating each resampling event 500 times.

There exist alternative measures of correlation. We explored the consistency of our findings to a different measure: the Spearman rank correlation coefficient. In this sensitivity analysis, we recalculated the correlation coefficients for both the observed data and the theoretical complete-synchrony scenario.

### Gravity style model

Gravity models can be used to describe population flows [11]–[13]. Here we used them to explore their ability to explain the correlation in the epidemic curves between pairs of village clusters:
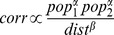
where *pop_1_* and *pop_2_* are the populations of the two settlements and *dist* is the Euclidean distance between the two settlements. By log-transforming the equation, we can estimate the exponents α and β through linear regression:
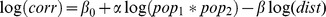
We used Akaike's Information Criterion (AIC) to compare the performance of the gravity model to an intercept only model and a univariate model incorporating Euclidean distance only (Table 2) [19]. All of the models were performed using the correlation coefficients from each set of down-sampled curves (500 in all). We reported the mean coefficient across all sets of down-sampled curves for each model. In addition we calculated 95% confidence intervals using the 2.5% and 97.5% quantiles from the distribution of coefficient estimates.

**Table 2:** Model summary.

Model 1	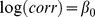
Model 2	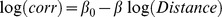
Model 3	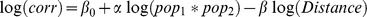

Overview of the different models used to estimate the correlation in the epidemic curves between pairs of village clusters in Kamphaeng Phet.

All analyses were conducted in R 2.15.2 [20].

## Results

Between 1994 and 2008, 4,768 dengue inpatients at KPPPH were successfully geocoded (93% of all cases) (Table 1) coming from 568 different villages (Figure 1). The provincial capital, where KPPPH was located, had 732 cases (15% of all cases). The mean age of cases was 11.0 years and 59% of cases suffered from the more severe hemorrhagic form of the disease (Table 1). On average, villages were separated by 1.4 km from their closest neighboring village.

**Figure 2:**
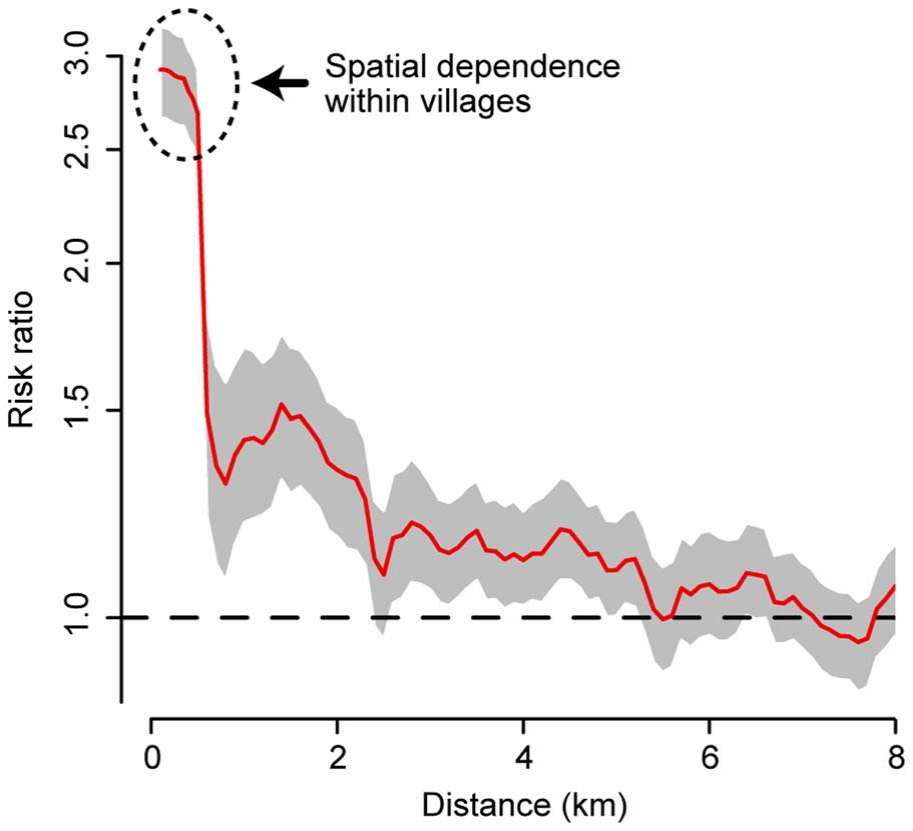
Short-term spatial dependence between cases. Spatial dependence between cases occurring within the same month as measured through *φ(d_1_, d_2_)* where *d_1_* and *d_2_* is the distance range between cases. The spatial range (*d_2_−d_1_*) was kept constant at 1 km when *d_2_* was greater than 1 km. When *d_2_* was less than 1 km, *d_1_* was equal to zero. Estimates are plotted at the midpoint of the spatial ranges.

We characterized the short-term spatial dependence between the home locations of the cases presenting at KPPH using the *φ(d_1_, d_2_)* statistic. We found that cases were 2.9 times more likely (95% confidence interval of 2.7–3.2) to occur both within the same community and to be infected within the same month of each other than the independent probabilities of occurring within the same community over the entire study period and occurring within the same month across the entire province (Figure 2). This fell to 1.4 times (1.2–1.7) for communities separated by between 0.5 km and 1.5 km and to 1.2 times (1.1–1.3) for communities separated by 2.5 km −3.5 km. We observed significant spatial dependence, albeit at low levels, at distances up to 5 km. However, when we divided the entire time series into smaller subsets covering five year time periods only, there was a clear trend in the spatial extent of spatial dependence (Figure S1). Cases from the 1990s exhibit spatial dependence at larger distances than more recent cases.

**Figure 3:**
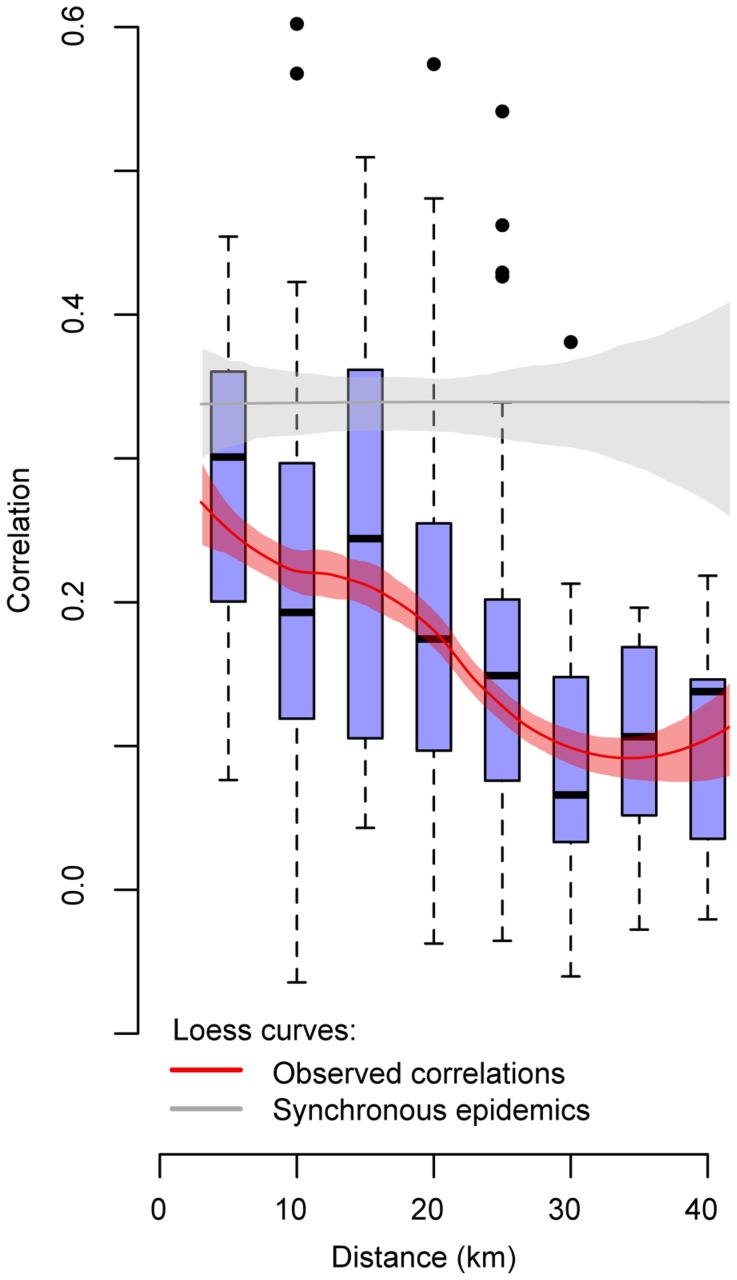
Correlation between epidemic curves. Box plots of the correlation between the epidemic curves of pairs of village clusters and the distance between them (blue). Only village clusters with at least 40 cases were used in this analysis. Loess curves of the same data with 95% confidence intervals generated through 500 bootstrapped resamples (red). The grey line represents the correlation under the theoretical scenario of complete synchrony in case distribution across the whole district (generated by randomly reassigning the dates that cases occurred between all the cases, keeping the total number at any time point fixed).

To explore whether short-term spatial dependence between individual cases resulted in similar patterns of disease observed between communities, we compared the correlation of the epidemic curves between communities by the distance separating them. We divided the villages into 24 village clusters with each village cluster having at least 40 cases over the 14 years. The locations of the village clusters are illustrated by the red dots in Figure 1. The mean correlation in the monthly epidemic curves between all village cluster pairs was 0.19, however, there existed substantial structure in the correlation: village clusters that were under 5 km apart had a mean correlation of 0.28 (95% confidence interval of 0.25–0.31), whereas village clusters separated by between 20 km and 25 km had a mean correlation of 0.16 (95% confidence interval: 0.14–0.17) (Figure 3).

We estimated that a (theoretical) scenario of complete synchrony across the entire province would result in a mean correlation of 0.32, irrespective of distance between village clusters (Figure 3). This correlation was much less than 1.0 as there are fewer cases than locations for many time points resulting in occasional small peaks in the epidemic curves that were not matched across all locations. The correlation under full synchrony and the observed correlations looked very similar when the alternative Spearman rank correlation coefficient was used instead (Figure S2).

We explored whether different statistical models could explain the observed correlation between community-pairs (Table 2). We found that univariate model incorporating only the Euclidean distance separating communities explained only 7% of the variance in the correlations (Table 3). Incorporating population sizes (model 3) substantially improved the fit of the model although the majority of the variance remained unexplained (R^2^ of 0.13). Model 3 was also strongly favored by AIC [21].

**Table 3:** Model coefficients.

	Intercept	Population	Distance	R^2^ (a)	AIC(a)
Model 1	0.16 (0.15–0.17)			-	643
Model 2	0.37 (0.29–0.45)		1.38 (1.27–1.50)	0.07	627
Model 3	0.11 (0.06–0.16)	1.22 (1.11–1.34)	1.09 (1.07–1.12)	0.13	610

Exponentiated coefficients estimates and 95% confidence intervals for the models set out in Table 2.

(a) Mean from 500 resamples.

## Discussion

We have used a large dataset from a long time series with geocoded addresses to explore the spatial patterns of dengue cases in a rural region with endemic circulation. We have shown substantial short-term clustering of dengue cases within communities, consistent with transmission chains circulating at small spatial scales. We observed a large drop in the clustering of cases from within-community to between community scales. Our findings suggest that upon discovering an infected individual, there is a significant risk that other individuals from his or her village will also be infected. The removal of mosquitoes in that community could potentially reduce the risk of onward transmission.

While lower than within-community estimates, significant short-term spatial dependence was nevertheless observed at inter-settlement scales. This observation is consistent with viral movements between neighboring communities, distances greater than the flight range of the dengue vector [10]. These findings point to a potential role for human movement in driving the spread of the virus. This was further supported by a clear reduction in the correlation in the epidemic curves between communities with increasing spatial separation between them. Gravity models are regularly used to describe human population flows [11]–[13]. Here a related formulation of gravity models that describes the correlation in the epidemic curves between communities was found to outperform competing models. This finding supports previous findings from gravity models fit to phylogeographic data from southern Vietnam [15]. Human movement has also been suggested to play a major role in the dengue epidemic in Iquitos, Peru [22]. Spatial correlation in ecological conditions (e.g., vector density) or in behavioral factors (e.g. the use of screens on windows) between communities may also explain these observations. We cannot definitively differentiate between these potential explanations here. Further research using information on the infecting pathogen, such as serotype or genetic information could help disentangle these competing hypotheses.

Our findings of focal patterns of disease support the results of previous cluster studies in the region [8], [9]. In addition, a previous study in Bangkok observed short-term spatial dependence in the homes of hospitalized cases between 1995 and 1999 at distances up to around 1 km [5]. Overall, we observed spatial dependence at larger distances than in the Bangkok study although when we looked at 5-year subsets of the data, the spatial extent of clustering was shorter among more recent cases. Higher levels of movement across the province as a whole suppresses spatial dependence by promoting the global mixing of the population. Our observations are therefore consistent with increased movement across the province in more recent years.

Mosquito control efforts are widely used throughout Southeast Asia and center on the use of insecticides. Insecticide fogging has been shown to temporarily reduce the number of mosquitoes in any location [23]. However, the ability of insecticides to reduce the risk of dengue infection remains unclear. Insecticide effectiveness may be limited by an inability to reduce mosquito density sufficiently or for a long enough period to prevent transmissions from viremic individuals. This is supported by a lack of a clear relationship between vector density and dengue transmission risk [24]. In addition, spraying may be too spatially restricted, allowing mosquitoes outside spray zones to rapidly repopulate fogged spaces. Finally spraying is sometimes only deployed in outdoor areas whereas *Aedes aegypti* mosquitoes tend to be found inside households. Estimating the impact of insecticides on dengue infection is difficult. The majority of dengue infections are not detected and the appropriate characteristics of control populations for any study are unclear. Nevertheless, further studies are needed to provide a sound evidence base for the widespread use of these measures.

The study has some limitations. The mean correlation between the epidemics experienced by pairs of communities appeared low (mean of 0.19). However, this was only slightly less than expected if all cases at any time point were randomly distributed throughout the communities (mean of 0.32), resulting in synchronous epidemics. This low level of correlation occurs because of the small numbers of cases (all the epidemic curves were down-sampled to only 40 cases). Even in the scenario of complete synchrony, tiny fluctuations were regularly present in the epidemic curve in one location and not in the curves of others, deflating correlation. These observations illustrate the problems in using the absolute correlation as a marker of similarity when many time points have no cases. Nevertheless, trends in correlation over distance and comparisons to a distribution expected under complete synchrony remain useful. Our data consists of cases that presented at hospital only. The majority of infections, however, result in asymptomatic or only mildly symptomatic. The spatial dependence between these infections may be different. We could only geocode individuals to the village level. We could not therefore explore spatial differences within any village. Future work using exact home locations may allow elucidation of finer scale spatial dependence between case homes. Finally, the relationship between gravity models fit to population flows directly and those fit to the correlation in epidemic curves may be complex and setting specific. Further work using simulated data may help provide insight into their relationship.

In conclusion, cases of dengue appear highly spatially correlated within villages in rural Thailand; however, neighboring communities nevertheless appear to observe correlated epidemics. Human movement patterns may be a key driver of dengue dispersal in this region. Future studies that incorporate movement diaries or GPS trackers would help describe population flows and allow the development of mechanistic models for the dispersal of dengue.

## Supporting Information

Figure S1 **Short-term spatial dependence between cases within 5-year windows.** Spatial dependence between cases occurring within the same month as measured through *φ(d_1_, d_2_)* where *d_1_* and *d_2_* is the distance range between cases. Individual estimates were generated using only cases from each 5-year window. The spatial range (*d_2_−d_1_*) was kept constant at 1 km when *d_2_* was greater than 1 km. When *d_2_* was less than 1 km, *d_1_* was equal to zero. Estimates are plotted at the midpoint of the spatial ranges.
(PDF)

Figure S2 **Correlation between epidemic curves using Spearman Rank coefficients.** Box plots of the correlation between the epidemic curves of pairs of village clusters and the distance between them as measured through Spearman Rank coefficients (blue). Loess curves of the same data with 95% confidence intervals generated through 500 bootstrapped resamples (red). The grey line represents the correlation under the theoretical scenario of complete synchrony in case distribution across the whole district (generated by randomly reassigning the dates that cases occurred between all the cases, keeping the total number at any time point fixed).
(PDF)
