# Supplementary figures and images for: The Spatial Dynamics of Dengue Virus in Kamphaeng Phet, Thailand
Source: PLoS Negl Trop Dis. 2014 Sep 11;8(9):e3138. doi: 10.1371/journal.pntd.0003138 (PMC4161352; doi:10.1371/journal.pntd.0003138)

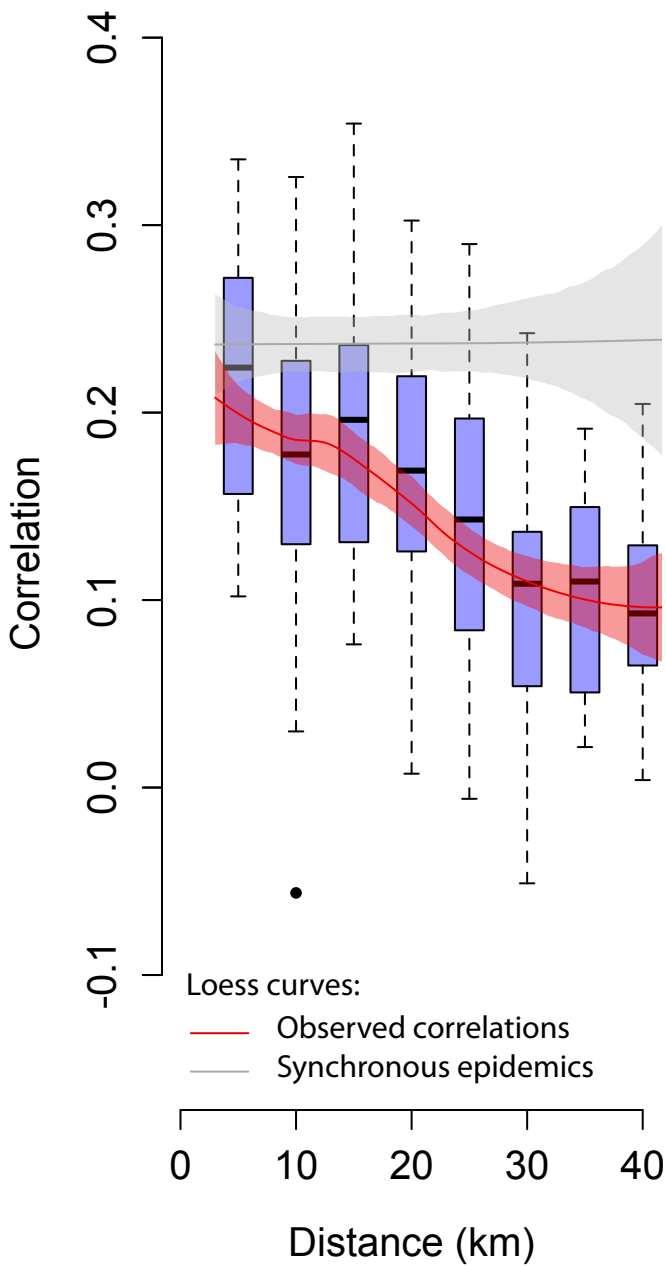

Supplement: Figure S1 — Short-term spatial dependence between cases within 5-year windows. Spatial dependence between cases occurring within the same month as measured through φ(d1, d2) where d1 and d2 is the distance range between cases. Individual estimates were generated using only cases from each 5-year window. The spatial range (d2−d1) was kept constant at 1 km when d2 was greater than 1 km. When d2 was less than 1 km, d1 was equal to zero. Estimates are plotted at the midpoint of the spatial ranges. (PDF) [file pntd.0003138.s001.pdf]

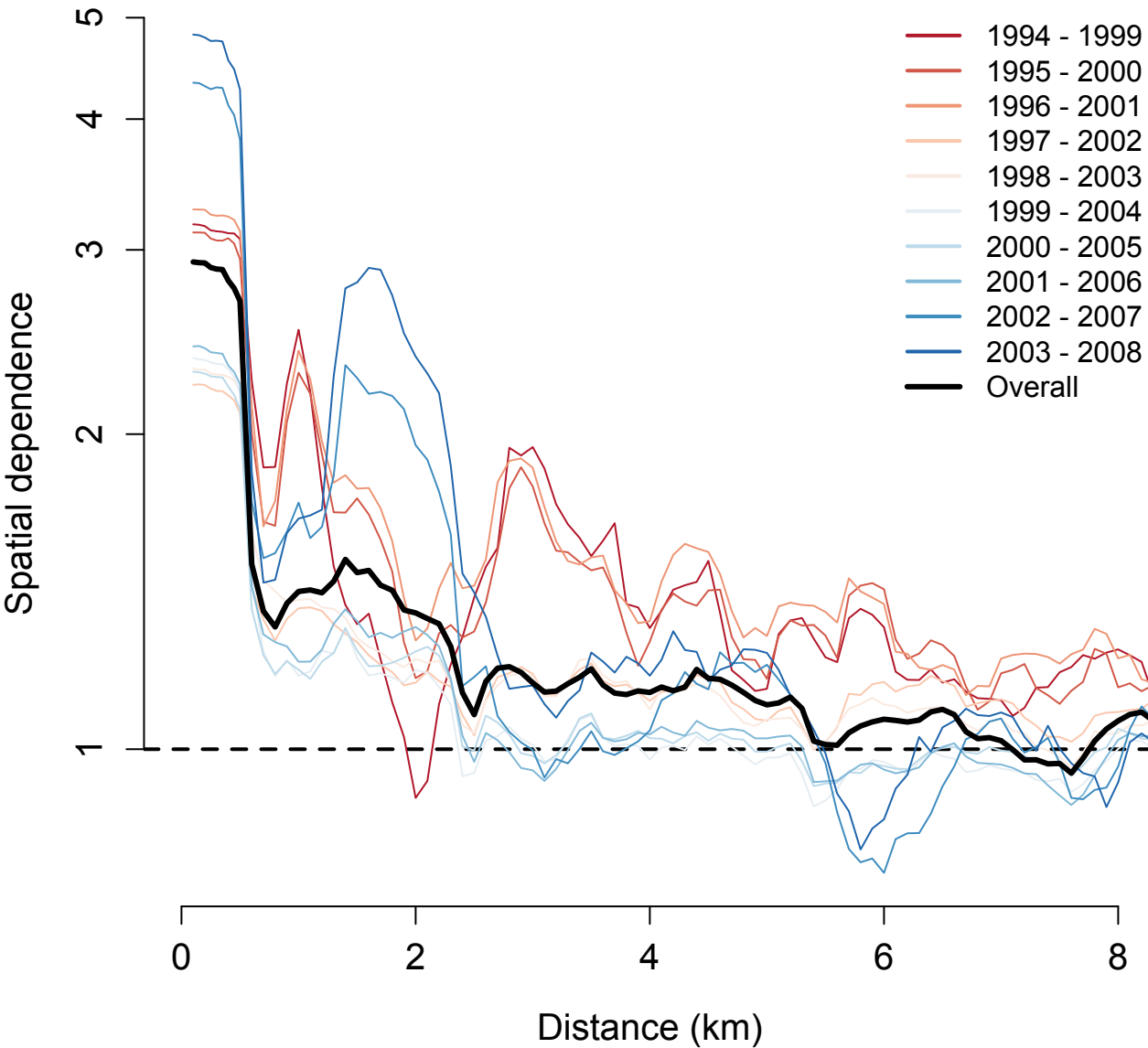

Supplement: Figure S2 — Correlation between epidemic curves using Spearman Rank coefficients. Box plots of the correlation between the epidemic curves of pairs of village clusters and the distance between them as measured through Spearman Rank coefficients (blue). Loess curves of the same data with 95% confidence intervals generated through 500 bootstrapped resamples (red). The grey line represents the correlation under the theoretical scenario of complete synchrony in case distribution across the whole district (generated by randomly reassigning the dates that cases occurred between all the cases, keeping the total number at any time point fixed). (PDF) [file pntd.0003138.s002.pdf]
